# Evaluation of a Telesimulation Program to Support Psychosocial Communication Training for Neonatology Fellows

**DOI:** 10.3390/bs16091600

**Published:** 2026-09-09

**Authors:** Daphne O. Darmawan, Virginia A. Marchman, Katarina Scala, LaTrice L. Dowtin, Weichen Ling, Shannon G. Hanson, Miller Shivers, Traci S. Williams, Keira Sorrells, Elisa B. Fuller, Sue Hall, Melissa Scala

**Affiliations:** 1 Department of Pediatrics, Stanford University School of Medicine, Stanford, CA 94305, USA; ddarmawan@stanford.edu (D.O.D.); wvling@stanford.edu (W.L.); 2 Department of Psychology, Stanford University and Department of Developmental Behavioral Pediatrics, Stanford School of Medicine, Stanford, CA 94305, USA; marchman@stanford.edu; 3 Department of Biology, University of California, Los Angeles, CA 90095, USA; kscala2003@gmail.com; 4 PlayfulLeigh Psyched, Silver Spring, MD 20910, USA; 5 Department of Pediatrics, Wake Forest University School of Medicine, Winston-Salem, NC 27157, USA; shannon.hanson@wfusm.edu; 6 Department of Psychiatry and Behavioral Health, Northwestern Feinberg School of Medicine, Chicago, IL 60611, USA; mshivers@luriechildrens.org; 7 Independent Researcher, East Point, GA 30344, USA; traciswilliams@gmail.com; 8 NICU Parent Network, Georgetown, IN 47122, USA; keira@nicuparentnetwork.org; 9 Secure Psychology, Los Angeles, CA 90019, USA; efuller@drfullerphd.com; 10 Independent Researcher, Ventura, CA 93001, USA; suehallmd@gmail.com

**Keywords:** medical education, mental health, communication, neonatology, digital platform

## Abstract

Parents of infants in the Neonatal Intensive Care Unit (NICU) frequently experience significant emotional distress, yet neonatology fellows receive limited training in psychosocial support. This study evaluates the use of online standardized-patient simulation to improve fellows’ communication skills addressing parent concerns. Fellows (n = 13) completed two virtual interactions with standardized parents, with feedback from a neonatal mental health professional (NMHP), a former NICU parent, and a neonatologist using narrative comments and the Gap–Kalamazoo Communication Skills Assessment Scale. Narrative data underwent thematic analysis, and a within-subjects, repeated measures ANOVA examined communication ratings; knowledge and self-efficacy scores were analyzed using descriptive statistics and paired t-tests. Communication scores differed significantly across raters (F(2, 24) = 6.2, *p* < 0.007). Fellow and faculty ratings were comparable (*p* = 0.37), whereas parent ratings exceeded fellow self-ratings (*p* = 0.005). NMHPs and parents identified more thematic codes than fellows (22/26 and 25/26 vs. 15/26), highlighting information delivery, mental health, relationship building, and communication. Fellows’ self-efficacy decreased after participation, (t(12) = 2.88, *p* = 0.02, d = 0.83). This decline, alongside positive feedback on the simulation’s relevance, reflects increased recognition of the complexity of addressing parental distress and underscores the need for ongoing training and real-world application. This study provides preliminary evidence for the feasibility of using online simulation to train neonatology fellows in communication strategies that support the psychosocial needs of NICU parents.

## 1. Introduction

### 1.1. Psychosocial Needs of NICU Parents

Parents of infants admitted to the Neonatal Intensive Care Unit (NICU) experience high levels of emotional distress, commonly reporting stress, guilt, shame, and symptoms of anxiety and depression (Roque et al., 2017; Shetty et al., 2024). Approximately 20% develop a diagnosable mental health disorder within one year of the infant’s birth, most frequently postpartum depression and post-traumatic stress disorder (Lefkowitz et al., 2010; McKeown et al., 2023). Although not all parents require formal mental health services, most benefit from enhanced emotional support (Hynan et al., 2013).

Parent emotional well-being directly affects engagement in infant care and the developing parent–infant relationship (Bernardo et al., 2021; Gerstein et al., 2019). Mothers of preterm infants experience higher stress than mothers of hospitalized term infants, and elevated stress has been associated with reduced capacity for decision-making and participation in infant care (Chourasia et al., 2013). Research on the experiences of non-birthing parents is limited and has focused largely on fathers in heterosexual families, restricting generalizability to the wide range of family structures represented in the NICU (Loewenstein, 2018; van Wyk et al., 2024). Available literature suggests that fathers often feel excluded from care and overlooked by staff leading to distress and isolation (Merritt, 2021).

NICU providers may inaccurately assume parents’ needs. Families marginalized by income, race, language, or insurance status are particularly vulnerable to insensitive communication, despite often disproportionately high needs for support (Bernardo et al., 2021; Sigurdson et al., 2020). These disparities underscore the central role of effective communication in family-centered NICU care (Davidson et al., 2007). Parents value timely, clear information about their infant’s condition and reassurance from NICU staff (Mundy, 2010). Because NICU admission disrupts the parental role, education and guidance on engaging in infant care may be particularly important to support bonding and improve outcomes (Craig et al., 2015).

### 1.2. Training in Providing NICU-Based Psychosocial Support

The Accreditation Council for Graduate Medical Education (ACGME) requires neonatology fellows to understand the emotional impact of the NICU experience, communicate effectively with families, and refer for behavioral or mental health concerns when indicated (Accreditation Council for Graduate Medical Education, 2025). Despite this mandate, education in mental health remains limited and has been identified as a high priority by the American Board of Pediatrics Strategic Planning Committee (McMillan et al., 2020).

This pilot study builds on prior work evaluating an online course that improved fellows’ knowledge and self-efficacy in recognizing and mitigating NICU family emotional distress (Scala et al., 2022). In that study, fellows completed self-directed modules aligned with the American Board of Pediatrics Road Map for mental and behavioral health education. The course, Caring for Babies: Advanced Provider Version (www.myperinatalnetwork.org), represents the first national pediatric family mental health program. Although effective in improving knowledge and self-efficacy, the prior study did not assess application of these skills in simulated clinical encounters. Gaps between cognitive understanding and clinical performance are well documented, highlighting the need for practice-based learning (Davis et al., 2006; Ghonein et al., 2019). The current study evaluates fellows’ implementation of learned skills in a simulated clinical environment with standardized patients.

### 1.3. Simulation-Based Medical Education

Standardized patients are often employed to teach communication skills in medical education (Linder et al., 2025; Rutherford-Hemming et al., 2024). In neonatology, simulation-based communication training has focused primarily on antenatal counseling, end-of-life discussions, and other high-stakes conversations (Janice-Woods Reed & Sharma, 2016; Munoz-Blanco & Boss, 2023). Despite this, a 2009 national survey found that 75% of graduating NICU fellows had not participated in role-play or simulation-based communication training (Boss et al., 2009). To our knowledge, literature specifically describing the use of standardized patients to teach trauma-informed communication in the NICU setting is limited, though similar approaches have been described in primary care and obstetric settings (Green et al., 2016; Olson et al., 2024). While prior telesimulation studies have demonstrated feasibility and acceptability for procedural and antenatal communication training, no published work has evaluated telesimulation specifically for teaching trauma-informed psychosocial communication to neonatology fellows, nor has prior work incorporated the perspectives of NMHPs or prior NICU parents. The present study addresses this gap by evaluating a multimodal telesimulation program that integrates interprofessional and family perspectives in both the simulation and assessment process.

Telesimulation, or simulation-based education delivered via videoconferencing, has emerged as a method to expand access to training. Its use accelerated during the COVID-19 pandemic, with early studies supporting its acceptability as a substitute for in-person instruction (Patel et al., 2020; Thomas et al., 2021). Telesimulation has since been adopted for procedural training, neonatal resuscitation, and communication skills development (Milede et al., 2021; O’Rae et al., 2021). Prior work has demonstrated the feasibility of teleconferenced standardized patient encounters for teaching empathic communication during antenatal consults (Kasat et al., 2020; Kim et al., 2021). Here, we present the results of a telesimulation training program for neonatology fellows.

## 2. Materials and Methods

### 2.1. Study Design and Participants

This pilot study used a mixed-methods, within-subjects pre–post design to evaluate an online simulation-based communication training program for neonatology fellows. Fellows were recruited from ACGME-accredited neonatology training programs in the United States. Eligible participants were current NICU fellows who had completed the Caring for Babies: Advanced Provider Version online mental and behavioral health curriculum prior to participation in the simulation. Participation was voluntary, and fellows were compensated with $50 Amazon gift cards upon simulation completion. The study was approved by the Institutional Review Board of Stanford University and subsequently the five other institutions from which fellows participated. This study received grant funding from the Association of Pediatric Program Directors.

Paid professional standardized patients were recruited from Stanford University and University of California, Los Angeles simulation programs and were trained to portray NICU parents after attending a one-hour orientation to the study’s aims and methodology. Standardized patients were provided with written scenario scripts including character background information and instructed to remain in character throughout the encounter while portraying a consistent level of emotional distress. They then rehearsed scenarios with a former NICU parent and NICU mental health professional (NMHP). Six NMHPs (five doctoral level psychologists and one maternal-infant psychiatrist) were recruited from the National Network of NICU Psychologists (www.nationalperinatal.org/psychologists; accessed on 3 February 2022) on a voluntary basis. Seven former NICU parents were recruited from the NICU Parent Network (www.nicuparentnetwork.org). NMHPs and former NICU parents attended a one-hour study orientation, including instruction on scoring of the Gap–Kalamazoo Communication Skills Assessment Form, hereafter referred to as the “Kalamazoo scale” (Peterson et al., 2014). The NMHPs, NICU parents, and neonatologists (study investigators) were not paid for their participation.

### 2.2. Simulation Intervention

Fellow participants were randomly assigned two of four available standardized patient scenarios. The four scenarios depicted diverse family structures and clinical contexts but were consistent in depicting a NICU parent experiencing emotional distress. Attention was paid to creating diversity of race, ethnicity, and sexual orientation in the scenarios. These design choices were intended to expose fellows to the range of family structures and backgrounds encountered in the NICU and to prompt culturally responsive communication. Each fellow entered a virtual breakout room via a secure videoconferencing platform and was joined remotely by an NMHP, former NICU parent, neonatologist, and standardized patient. Observer composition varied across simulation scenarios in order to reduce rater familiarity effects. The NMHP gave the fellow a brief introduction to the patient and family, and instructed the fellow to address the standardized patient’s concerns over 15 min while the remaining observers turned off their cameras. Each simulation depicted a common NICU scenario involving a parent experiencing emotional distress related to their infant’s hospitalization as opposed to addressing critical “high-stakes” or palliative care scenarios (Supplemental Materials A).

Each simulation session was followed by a structured debriefing session facilitated by an NMHP as instructed by the study team to include reflective discussion, targeted feedback on communication strategies, and opportunities for fellows to identify strengths and areas for growth. Former NICU parents, neonatologists, and standardized patients were invited to share their experiential perspectives during a dedicated portion of the debrief. All debriefs followed the same sequential structure: fellow self-reflection, NMHP feedback, parent/standardized patient perspectives, and facilitator synthesis. Once the first simulation session and feedback session were complete, the fellow moved to a second breakout room and repeated the experience with a different scenario, standardized patient, and observers.

### 2.3. Measures and Data Collection

Prior to participating in the simulation exercises and after completing the “Caring for Babies” curriculum, each fellow completed a baseline knowledge assessment about understanding of mental health issues (Scala et al., 2022). Knowledge questions reflected four domains (8 questions each): Trauma Informed Communication, Infant Mental Health, Parent Mental Health, and Comprehensive Mental Health. Cronbach’s alpha scores indicated acceptable internal consistency at both the pre- (α = 0.79) and post-simulation (α = 0.79) assessments. Fellows also completed pre- and post-simulation self-efficacy surveys assessing confidence in addressing NICU parents’ emotional distress. Three domains were represented (8 questions each): Communication, Infant Mental Health, Parent Mental Health (Supplemental Materials B). Self-efficacy items were rated on a Likert scale (1 = strongly disagree, 6 = strongly agree). Scores within each scale were averaged to generate a mean score per fellow for each rater with higher scores indicating greater perceived confidence. Cronbach’s alpha scores suggested weaker internal consistency scores at pre-simulation (α = 0.50), but good internal consistency at post-simulation (α = 0.90). NMHPs and former NICU parents were blinded to the fellows’ self-efficacy assessment scores.

Communication skills were assessed using the Kalamazoo scale, a validated instrument designed to evaluate clinician-patient communication behaviors. The assessment form contains Likert scale (1 = poor, 5 = excellent), forced-choice, and free-text fields that identifies evidence-based “essential elements” with associated skill competencies for effective physician-patient communication (Rider & Nawotniak, 2010). Key components of physician communication were represented across 9 domains: (1) builds a relationship, (2) opens the discussion, (3) gathers information, (4) understands patient and family perspective, (5) shares information, (6) reaches agreement, (7) provides closure, (8) demonstrates empathy, and (9) communicates accurate information (Joyce et al., 2010; Peterson et al., 2014). Following each simulation, fellows completed self-assessments using the scale, while NMHPs and former NICU parents independently rated fellow performance. As the neonatologist study investigators held logistical responsibilities, Kalamazoo Scales were not completed by those observers and are therefore not included in the quantitative analyses. Scores for each assessment were averaged to generate an average score per scale/rater, with higher scores indicating better communication skills. Cronbach’s alpha scores indicated excellent internal consistency for all raters (α = 0.93–0.97). Narrative feedback was collected from all raters using standardized open-ended prompts embedded in the Kalamazoo assessment form asking about effective communication behaviors that were observed and specific suggestions for improvement. No word limit was imposed on responses. Individual rater identities within each group were not linked to specific comments during analysis.

### 2.4. Data Analysis

For the knowledge, self-efficacy, and communication assessments, all scores were averaged over multiple responses per scale (>5) and rater, yielding complete data for all participants. Shapiro–Wilks tests were performed to examine whether the resulting scores were normally distributed; all scales met this assumption (all *p* > 0.11), except for efficacy scores pre-simulation. Since the resulting distributions generally approximated a continuous, interval scale, central tendencies and rater differences were analyzed using descriptive and inferential parametric statistics that are appropriate for interval-scale data. However, given the small sample size and that one scale was not normally distributed, statistical results should be interpreted with caution and as primarily descriptive.

We first explored whether mean knowledge and self-efficacy scores were different for those fellows who participated in the simulations from other eligible fellows. Between-group t-test statistics are reported, but should be interpreted with caution given the unequal sample sizes. Our main analyses focused on the impact of the simulation exercise on self-efficacy and communication ratings. To determine if self-efficacy scores were different pre- and post-simulation, we conducted within-subjects t-tests, reporting effect size as Cohen’s d. A within-subjects analysis of variance (ANOVA) using planned comparisons with Tukey correction was used to examine differences in Kalamazoo scale communication scores across raters (fellows, NMHPs, and former NICU parents). All levels of significance were set at *p* < 0.05, two-tailed. Exploratory analyses further examined the concordances using Pearson correlations between raters on the communication assessment, as well as between the baseline knowledge and self-efficacy assessments. While our small sample size precludes interpretations based on statistical significance for these associations, we interpret our findings in terms of effect sizes as r > 0.30 and higher.

Qualitative narrative data were analyzed according to the Braun and Clarke thematic analysis framework using an inductive approach (Braun & Clarke, 2006). The coding team consisted of four individuals with diverse professional backgrounds, one neonatologist and three NMHPs, each with at least doctoral level experience in qualitative studies, whose varied positionalities were considered an asset in identifying a broad range of communicative behaviors across clinical and experiential frames of reference. The coders independently reviewed all comments before convening for iterative consensus meetings in which interpretive assumptions were surfaced, discussed, and documented, serving as a form of reflexive peer debriefing. Thematic saturation was considered achieved when iterative review of additional narrative comments no longer generated new codes. Formal inter-coder reliability statistics were not calculated and instead discrepancies were resolved through consensus.

## 3. Results

### 3.1. Participants

Thirteen NICU fellows from five ACGME-accredited training programs completed both simulation encounters and all associated assessments. All participants had previously completed the Caring for Babies: Advanced Provider Version online mental and behavioral health curriculum. Six male and seven female fellows participated; a greater proportion were male compared to the pool of fellows eligible to participate in the study (81.5% female, χ^2^ = 11.2, *p* = 0.004). Fellows represented a range of postgraduate training years, with seven first-year fellows, five second-year fellows, and one third-year fellow.

Mean knowledge assessment scores (% correct) at baseline for participating fellows (M = 64.2%, SD = 14.2) were not significantly different compared to non-participating fellows (M = 67.2%, SD = 8.4), indicating that fellows participating in the online simulation had knowledge representative of the larger group of eligible fellows who had all completed the Caring for Babies course.

### 3.2. Self-Efficacy Outcomes

**Figure 1:**
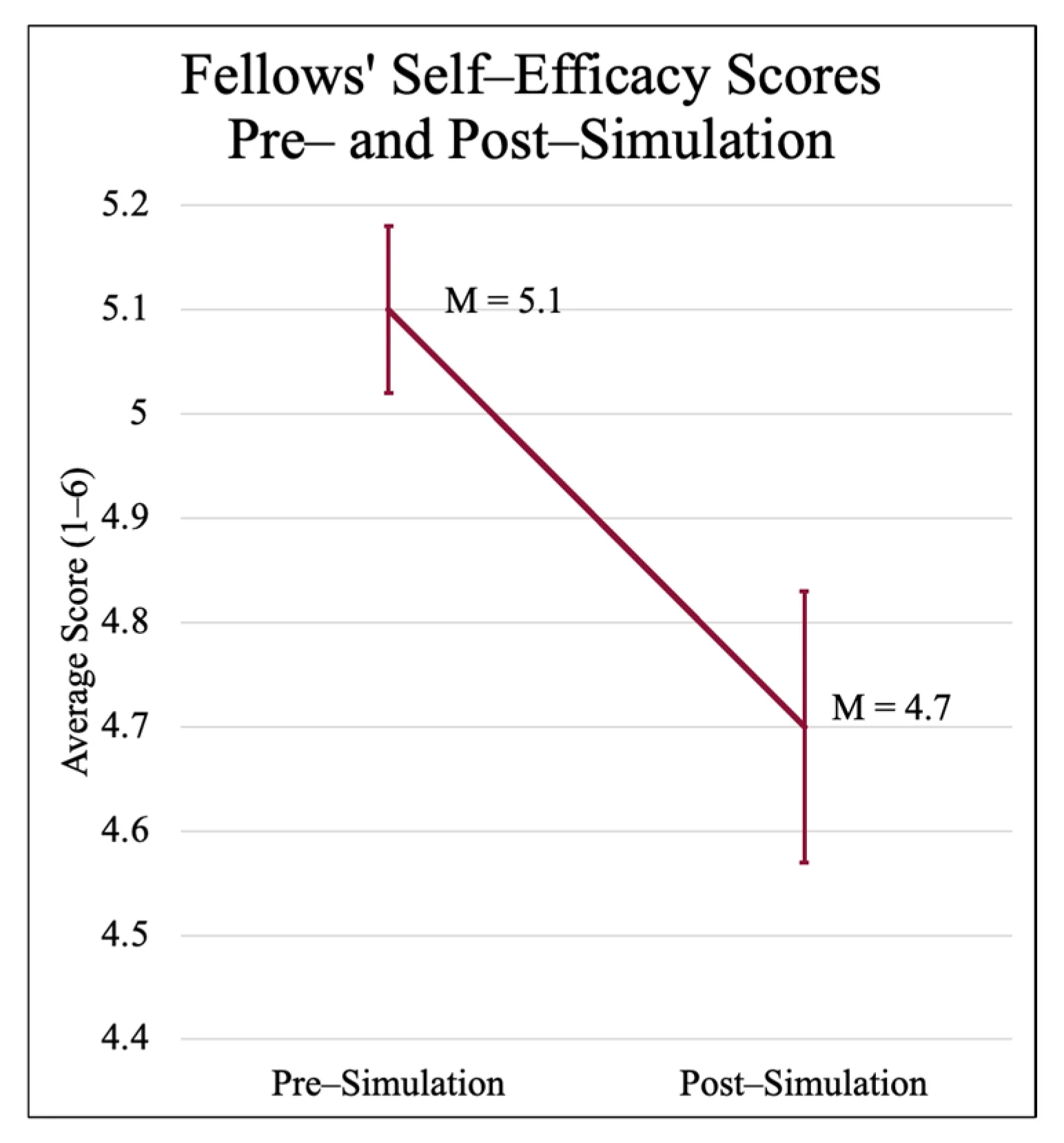
Fellows’ mean self-efficacy scores pre- vs. post-simulation on a Likert scale (1 = strongly disagree to 6 = strongly agree).

Baseline mean self-efficacy ratings were slightly lower for the participating fellows (M = 5.1, SD = 0.28, range 4.7–5.8) compared to the non-participating fellows (M = 5.2), although this difference did not achieve statistical significance. For those fellows who participated in the simulation program, self-efficacy ratings were significantly lower following their participation (M = 4.7, SD = 0.49, range 3.7–5.5) compared with baseline, t(12) = 2.88, *p* = 0.02, d = 0.83, CI: 0.08–0.60, suggesting that participating in the simulation exercise impacted fellow ratings of self-efficacy (Figure 1).

### 3.3. Communication Skills Assessment

**Figure 2:**
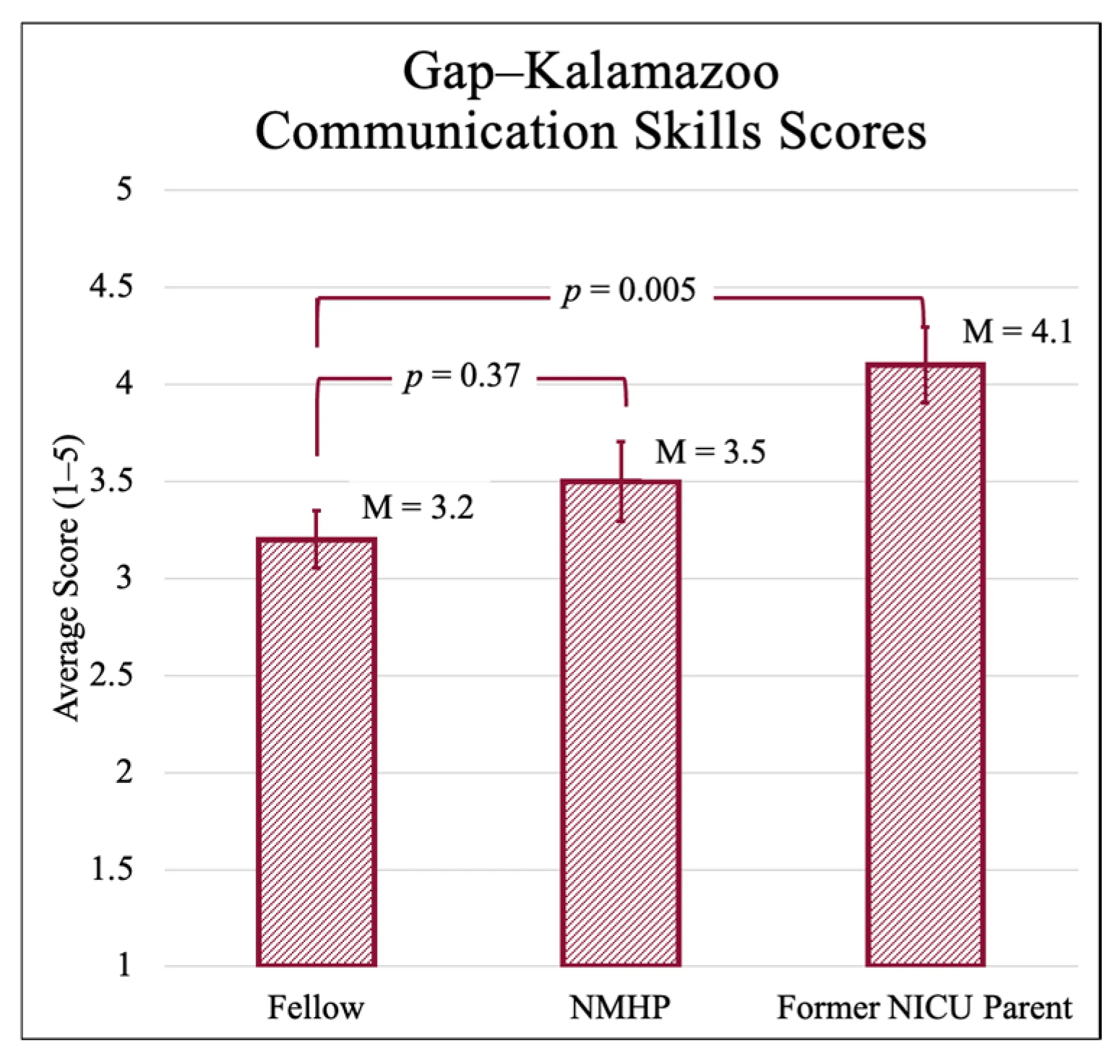
Fellows’ mean Gap–Kalamazoo Communication Skills scores on a Likert scale (1 = poor to 5 = excellent) as determined by fellow (self) vs. NMHP vs. former NICU parent.

Overall scores on the Kalamazoo communication scales reflecting fellows’ communication skills averaged between “good” and “very good” across all reporters. However, mean scores significantly differed depending on reporter, reflected in a significant main effect, *F*(2, 24) = 6.2, *p* < 0.007. Planned comparisons indicated that ratings by the fellow (M = 3.2, SD = 0.53, range = 2.1–3.9, CI: 2.9–3.6) and NMHP (M = 3.5, SD = 0.74, range 2.4–4.7, CI: 3.2–3.9) were generally comparable (t(24) = 1.4, *p* = 0.37, d = 0.47). Ratings by the former NICU parent (M = 4.1, SD = 0.7, range 2.8–5.0, CI: 3.6–4.4) were generally higher than by the fellow (t(24) = 3.5, *p* = 0.005, d = 1.24) (Figure 2).

### 3.4. Concordances Among Raters in Communication Skills and Among the Knowledge, Self-Efficacy, and Communication Assessments

Pearson correlations were used to explore the concordance among the raters on the Kalamazoo scales of effective communication. Results indicated a positive association between fellows and parents. Fellows who rated themselves more highly in communication were also rated more highly by parents (*r* = 0.39). However, there was less agreement between the ratings by fellows and clinicians. That is, fellows who rated themselves more highly in communication were not necessarily those who were rated highly by the NMHP (*r* = 0.11).

Exploratory correlational analyses also revealed that fellows who scored higher on the pre-simulation knowledge assessment were not necessarily rated higher on communication by themselves (*r* = 0.04), nor by the parent (*r* = 0.16) or the NMHP (*r* = −0.27). However, there was some suggestion that higher ratings of self-efficacy prior to participating in the simulation were weakly associated with higher communication scores by the fellow (self) (*r* = 0.36), but not by parents (*r* = 0.26) or NMHP (*r* = 0.29). Again, these correlations should be interpreted with caution given the small sample size.

### 3.5. Qualitative Feedback and Thematic Analysis

Four themes emerged from narrative feedback provided by fellows, NMHPs, and former NICU parents. The four themes and 26 relevant codes included (1) information delivery (medical information, NICU policies, available resources), (2) mental health (parent mental health, emotional regulation, bonding, and interaction with baby), (3) relationship building (empathy, compassion, rapport, trust, support, and shared decision making), and (4) communication (eliciting concerns, listening, acknowledging experience, timing of discussion, language used, behavioral techniques, limited time in simulation/running out of time, and communication which emotionally triggered observers). Themes with definitions and sample quotes from written feedback are presented in Table 1. Across all rater groups, most comments focused on themes of relationship building and communication. The most frequently reported code among fellows was eliciting concerns (16%). The most frequently reported codes among NMHPs were acknowledging the parent experience (13%) and empathy (12%). The most frequently reported codes among former NICU parents were empathy (9%) and rapport (9%). While NMHPs drew on clinical frameworks to identify systemic gaps (e.g., one NMHP noted, “Dads are also at risk of symptoms of depression, traumatic stress, and anxiety—not just mothers.”), former NICU parents responded to the emotional quality of the interaction, with one parent describing a fellow’s question “What are you thinking right now?” as particularly meaningful. Fellows’ self-reflections were comparatively sparse and tended toward behavioral self-critique (e.g., “I did not explore reasons for mother’s distress”) rather than systemic awareness. This contrast suggests that fellows may benefit from exposure to the experiential perspectives of former NICU parents to develop a more integrative understanding of the mental health needs of NICU families.

**Figure 3:**
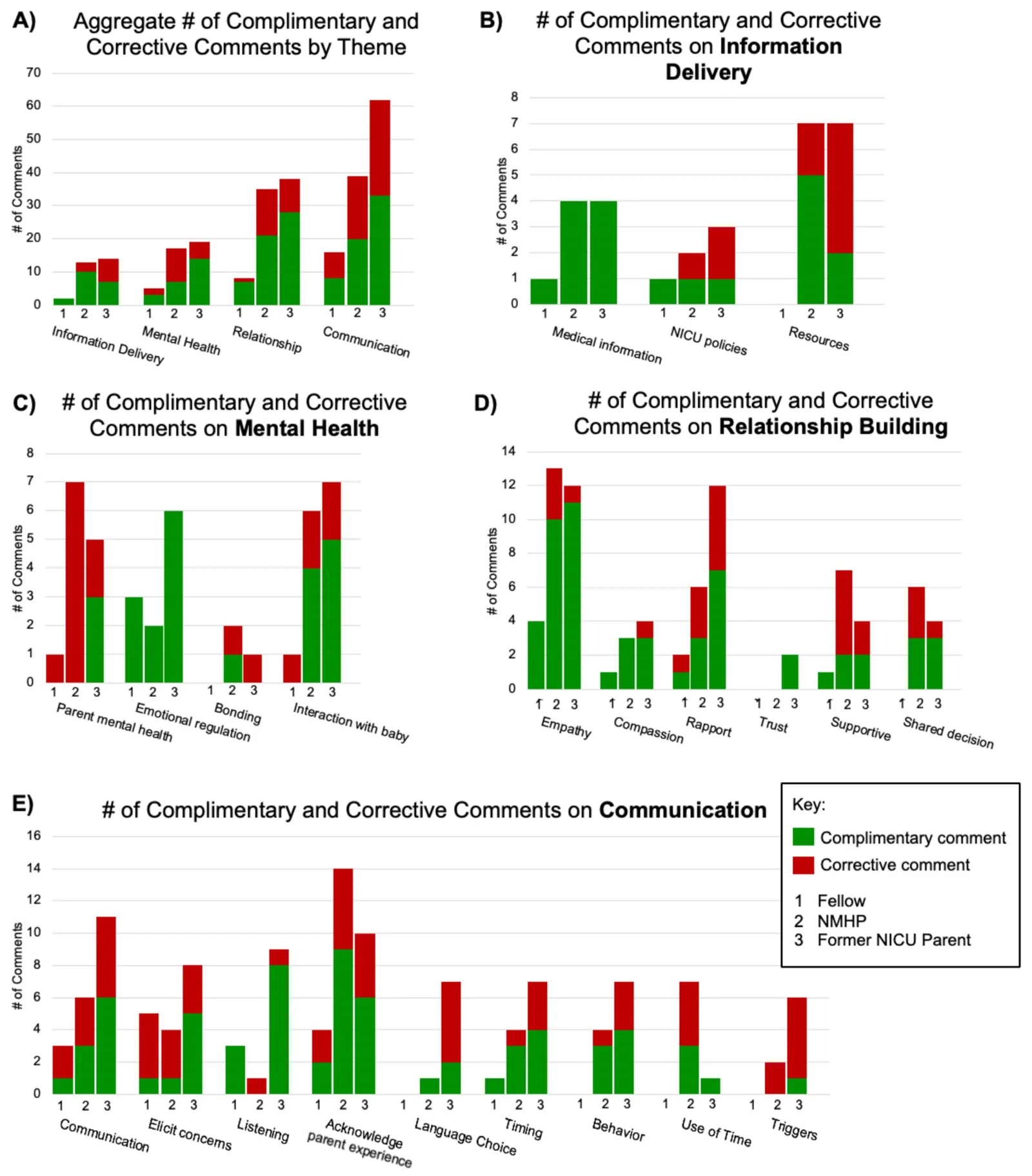
Number of complimentary and corrective narrative comments from qualitative analysis categorized by themes. (**A**) Aggregate number of narrative comments by themes. (**B**) Number of narrative comments on information delivery. (**C**) Number of narrative comments on mental health. (**D**) Number of narrative comments on relationship building. (**E**) Number of narrative comments on communication.

NMHPs and former NICU parents identified a greater number of thematic codes than fellows’ self-reflections (22/26 and 25/26 vs. 15/26 codes, respectively, with the denominator reflecting the total number of unique codes identified across all three rater groups as listed above). Of comments by fellows, 65% were complimentary of their performance compared to 55% of NMHPs and 60% of parent comments (Table 2). Fellow comments were more likely to center around their communication (40% of comments) compared to 29% of NMHP and 36% of parent comments. Similar percentages of comments were found in relationship (25%, 23%, and 27% for fellow, NMHP, and parent, respectively) and mental health domains (15%, 13%, and 13%). However, around information delivery, NMHP and parents were somewhat more likely to comment (6%, 11%, and 9%) but positive comments differed. All fellow comments about information delivery were complimentary of their performance while only 76% of NMHP and 50% of parent comments were complimentary in this area (Figure 3).

### 3.6. Fellows’ Feedback of the Simulation Experience

All thirteen fellows were asked why the simulation experience did or did not improve their ability to provide emotional support to NICU families. Responses included seven fellows who found it valuable for learning practical techniques in interacting with distressed parents. Two fellows emphasized the importance of practicing mental health support skills and noted that feedback from NMHPs and former NICU parents was particularly valuable compared with feedback from neonatologists alone. One fellow highlighted the realism of scenarios, and another noted that the simulations underscored the neonatologist’s role as a counselor in the NICU.

When asked whether the simulation would change their practice, the most common response was an expectation or hope that it would improve skills and had identified areas for growth. Three fellows reported gaining additional tools to support families, three cited the positive impact of hearing from the former NICU parent perspective, and three felt the experience would lead them to pay closer attention to families and approach them with greater empathy.

All fellows indicated they would recommend the simulation to colleagues. Six felt the skills addressed were highly important, including two who emphasized the value of practicing in a low-stakes learning environment. Three highlighted the benefit of immediate feedback, and two noted the usefulness of practical tools for supporting parents. One fellow commented on the uniqueness of the program compared with offerings at most fellowship programs.

## 4. Discussion

This pilot study demonstrates that an online, standardized patient-based simulation program is a feasible and effective method for training neonatology fellows in communication strategies to support the psychosocial needs of NICU parents. Fellows rated their own communication lower than NMHPs or parents did with parents assigning the highest scores. During post-session debriefings, parents praised fellows’ communication skills and reflected on their own prior experiences of poor communication with NICU providers. Concordance between fellows’ self-assessments and ratings by NICU parents or NMHPs on the Kalamazoo scale was weak, suggesting that different raters bring distinct perspectives and that fellows may lack awareness of their communication strengths and deficits. There was also little association between pre-simulation knowledge scores and observed communication effectiveness, indicating that content mastery did not predict communication performance. Fellows with higher pre-simulation self-efficacy scores rated themselves as more effective communicators, but were not necessarily rated higher by external observers, suggesting a disconnect between confidence and accurate self-appraisal. This may reflect individual personality traits or limited prior feedback on communication skills, leading to reduced self-awareness. This may also reflect systematic biases within sets of raters, rather than true differences in effective communication skills. Given the central role of effective communication in trauma-informed care, these findings underscore the need for enhanced training focused on fellow–parent interactions.

Self-efficacy scores for recognizing and mitigating emotional distress decreased from pre- to post-simulation. After practicing these skills, fellows reported lower confidence, likely reflecting increased awareness of previously unrecognized gaps in their knowledge and abilities. Lower fellow communication self-ratings combined with the qualitative finding that fellows identified fewer thematic codes than NMHPs or parents can be interpreted as potentially limited self-awareness in psychosocial communication. This finding aligns with prior studies demonstrating limited physician ability to accurately self-assess skills compared with external evaluators (Davis et al., 2006). However, we acknowledge that alternative interpretations are plausible. The observed decline in self-efficacy could reflect transient demoralization or reduced confidence in response to corrective feedback, rather than genuine improvement in self-assessment accuracy. This distinction would need to be resolved with an objective, external measure of communication skill in clinical practice. This finding is consistent with literature on self-assessment calibration in medical education, including studies demonstrating that novice learners may overestimate their abilities prior to exposure to structured feedback (the Kruger–Dunning phenomenon), and that feedback-induced reductions in self-efficacy may be a temporary, adaptive response that precedes longer-term skill development (Davis et al., 2006; Kennedy et al., 2004). Discrepancies between perceived competence and actual performance are well described in medicine. For example, Bibl et al. found that although neonatologists reported improved communication skills after a workshop, these gains were not reflected in observed performance (Bibl et al., 2021). Importantly, if the observed decline in self-efficacy reflects demoralization rather than calibration, it may represent an undesirable short-term outcome that requires intentional follow-up, including mentorship, reinforcing positive feedback, and longitudinal opportunities to practice these skills in supervised clinical settings.

Medical educators continue to explore effective strategies for developing clinical excellence, which likely requires a multi-pronged approach combining knowledge acquisition with performance assessment in both simulation and clinical settings, supported by high-quality, targeted feedback. Although most medical education simulations rely on expert-led group debriefing, (Gardner, 2013; Sawyer et al., 2016) this model may be limited for addressing mental health concerns, particularly given NICU fellows’ minimal prior mental health training (Scala et al., 2022). To address this gap, our study incorporated feedback from three expert perspectives: NMHPs, former NICU parents, and neonatologists, delivered through group discussion and written evaluations. We observed fewer reflective themes in fellows’ self-assessments than in feedback from NMHPs and parents, highlighting the unique value of these perspectives and the importance of their inclusion in mental health-focused educational programs.

In our study, fellows reported decreased confidence in conducting psychologically supportive conversations after the simulation, suggesting a need for additional education, clinical experience, and mentoring. Because most neonatologists have not received formal training in recognizing and mitigating emotional distress, they may be unaware of limitations in role modeling or in providing feedback on psychosocial aspects of care. Future educational strategies could include routine feedback from social workers or other NMHPs during fellow–parent interactions. Another approach would be to incorporate discussion of family psychosocial issues into daily rounds, treating it as a core “system” akin to nutrition or respiratory support, to normalize and elevate this aspect of care. Current models that limit family discussions to weekly multidisciplinary rounds may compartmentalize and inadvertently deprioritize psychosocial support. Given that NMHPs and parents identified relationship building as a deficit, assigning longitudinal fellow–family relationships, similar to primary nursing models, may be another strategy worth exploring. Further study of these and other interventions to support family mental health is needed.

Attention to diversity was a deliberate feature of our simulation design, with scenarios depicting families of varied race, ethnicity, sexual orientation, and family structure. Nevertheless, cultural responsiveness in NICU communication extends beyond family structure to include language concordance, health literacy, and navigation of systemic inequities. Future iterations of this training program could incorporate scenarios involving families with a primary language other than English, situations requiring interpreter services, or families facing socioeconomic barriers, as these contexts present unique communicative challenges that are not fully captured by the current simulation design. Expanding cultural responsiveness training in this manner would align with growing calls for equity-centered medical education and may be particularly important given documented disparities in NICU family communication and support.

This study has several limitations. As a pilot study, it included a small number of fellows, all NMHPs and former NICU parents were uncompensated volunteers, and not all participants completed post-simulation evaluations, particularly free-text responses. It is possible that parents with particularly negative prior experiences, and therefore lower expectations, were more likely to volunteer to participate in this study. Effects were evaluated only with fellows who were previously exposed to the “Caring for Babies” curriculum, limiting generalizability. Baseline data on prior simulation experience and other psychosocial training exposure were not collected, limiting the evaluation of the simulation as a standalone intervention. Future studies should incorporate these variables at baseline to allow for more nuanced interpretation of training effects. In addition, fellows may have performed better in the simulated setting than in clinical practice because the simulation allowed focused attention on family communication without competing clinical demands. Another limitation of the study is that inter-rater reliability following training on the Kalamazoo scale was not assessed. No formal calibration exercise with inter-rater agreement statistics was conducted prior to the study session. This limits our ability to attribute rater differences to true variation in communication behaviors versus systematic rater bias. Additionally, formal inter-coder reliability statistics were not calculated. Instead, discrepancies among coders were resolved through iterative consensus, which, while a recognized approach in qualitative research, precludes formal reliability reporting. While this simulation expands available educational programming, its impact on actual clinical performance was not measured. Prior studies have demonstrated mixed effects of communication simulations on performance and have shown that learning gains may diminish over time (Bibl et al., 2021; Blackmore et al., 2018; Green et al., 2016). However, neonatal fellows have previously reported that simulation is more effective than didactic instruction for managing difficult family conversations, while mentor observation and clinical practice are perceived as the most important learning methods (Lechner et al., 2016). Studies recommend repeated communication skills training, either in formal settings or embedded within clinical care, to achieve sustained improvement (Bibl et al., 2021; Ghonein et al., 2019).

## 5. Conclusions

Although medical education governing bodies mandate training in psychosocial and mental health support for patients and families, such instruction remains limited in neonatology training. This pilot study demonstrates the feasibility and perceived relevance of a telesimulation program designed to support neonatology fellows’ communication addressing NICU parents’ concerns, while expanding the limited educational offerings available to prepare trainees for the psychosocial dimensions of NICU care. Narrative feedback from NMHPs and former NICU parents identified key domains of effective psychosocial communication that were less frequently recognized by fellows themselves. Although fellows were more critical of their own communication skills and provided less detailed self-reflection, the inclusion of NMHPs and former NICU parents added critical depth and authenticity to training in psychosocial support, an area in which formal, adequate instruction is uncommon. The observed decrease in fellows’ self-efficacy following participation in the simulation, alongside positive qualitative feedback, likely reflects increased awareness of the complexity of addressing parental distress rather than diminished ability. Taken together, the quantitative and qualitative findings show that fellows underestimate their own strengths relative to how parents perceived them, and showed limited self-reflective range compared to NMHPs and parents. This pattern suggests that telesimulation incorporating interprofessional and parent perspectives may help calibrate self-assessment and highlight areas for growth in psychosocial communication, though the small sample size and pilot design preclude firm conclusions about generalizability. Further research is needed to determine how such interventions can be integrated longitudinally and translated into sustained clinical skill development in supporting NICU family mental health.

## Figures and Tables

**Table 1:** Themes identified in narrative feedback with definitions and representative quotes from each group.

Information Delivery: Fellow’s performance in the area of delivering medical information including sharing NICU policies and available resources to the NICU parents.	
Fellow	“I think I tried to offer a plan that would help alleviate some of the concerns (care conference, mental health resources).” “I explained the shifting schedules okay.”
NMHP	“You balanced providing medical advice, empathy and resources very well.” “Your decision to address the medical event by explaining in plain terms why the chest tube was placed helped de-escalate Mom’s most important concern in the moment.”
Former NICU Parent	“This doctor could have provided more specifics about NICU parent peer mentorship.” “He made sure to ask about bringing in additional services for the family.”
Mental Health: Fellow’s ability to consider and incorporate the parent’s mental health, bonding and interaction with the baby, which supports the infant’s mental health, and to support their own emotional regulation.	
Fellow	“We didn’t talk too much about the baby’s condition.” “I did not explore reasons for mother’s distress beyond the circumstances of the moment. In the future, would directly ask mother how she is handling the stress of the NICU.”
NMHP	“Dads are also at risk for symptoms of depression, traumatic stress, and anxiety—not just mothers. Suggesting conjoint psychotherapy focused on self-care and managing the stress of a NICU stay (not mental illness, per se) is a way to get dad the support he needs while he is actively doing something for/with his wife.” “Ask about baby as a person. Fellow could highlight that they know everything medical about the baby but haven’t had a moment to get to know baby in person, could follow up with asking how family is making their way through other care routines, diapers, bathing, etc.”
Former NICU Parent	“I loved the doctor asking the question ‘what are you thinking right now?’ and her advice for self-care..” “I do think offering some additional resources to the parent would have been good—financial counseling from billing to help with insurance issues dad was stressed about and perhaps support for the mom who was dealing with a lot of depression.”
Relationship Building: Fellow’s ability to express empathy, compassion, build and maintain rapport and trust, provide support and elicit shared decision making.	
Fellow	“I felt as though I established rapport through active listening and acknowledging the father’s frustration.” “I did not do much to build a relationship.”
NMHP	“Your ability to empathize with this very distressed and fearful parent was clear as evidenced by her sharing more with you about her fear, persistent worry, and thoughts of guilt/self-blame.” “Really did a great job of building rapport throughout the encounter, validating mom’s feelings.”
Former NICU Parent	“This fellow’s approach at building a relationship did not start off great but ended up having a really great recovery, allowing for an automatic building of a relationship.” “Fellow showed immense levels of empathy and human connection. Every family should be lucky to be cared for by this Neo Fellow.”
Communication: Fellow’s ability to effectively use language, engage in nonverbal communication, and utilize time.	
Fellow	“I want to find better ways to ask questions.” “I could have asked what they would like to do rather than offering my answers.”
NMHP	“You came across confident, calm, and reassuring, particularly your calm and consistent rate of speech.” “It is helpful to invite parents to sit or ask if you may join them sitting. This changes the perception of power dynamics and conveys a supportive interpersonal stance, as we might do with a friend.”
Former NICU Parent	“I thought this fellow did an excellent job at opening the discussion by changing up the setting. Moving to neutral environment shifted the mood, and allowing the ability to open up the dialog.” “’It’s going to depend on him/her (the baby)’ is a trigger for me and causes feelings of uncertainty in me”

**Table 2:** Proportions of complimentary feedback by reporter (fellow vs. NMHP vs. former NICU parent) across different themes.

	Information Delivery	Mental Health	Relationship Building	Communication
Fellow	100%	60%	87%	50%
NMHP	76%	41%	60%	51%
Former NICU Parent	50%	73%	73%	53%

## Data Availability

The original contributions presented in this study are included in the article/Supplementary Material. Further inquiries can be directed to the corresponding author.

## Supplementary Materials

The following supporting information can be downloaded at: https://www.mdpi.com/article/10.3390/bs16091600/s1, Supplemental Materials A: Simulation Scenarios; Supplemental Materials B: Self-Efficacy Assessment of Neonatal Fellows for the Study on “Caring for Babies and their Families: Providing Psychosocial Support in the NICU” Educational Course.

## Abbreviations

The following abbreviations are used in this manuscript:

NICU	Neonatal Intensive Care Unit
NMHP	Neonatal mental health professional
ANOVA	Analysis of variance
ACGME	Accreditation Council for Graduate Medical Education
