# Supplementary material for: Evaluation of a Telesimulation Program to Support Psychosocial Communication Training for Neonatology Fellows
Source: Behav Sci (Basel). 2026 Sep 9;16(9):1600. doi: 10.3390/bs16091600 (PMC13603085; doi:10.3390/bs16091600)
Supplement: Supplementary file 1 [file behavsci-16-01600-s001.zip › behavsci-4307090-supplementary.pdf]

Supplemental Materials:

Supp A. Simulation Scenarios
- Standardized Patient #1
- Standardized Patient #2
- Standardized Patient #3
- Standardized Patient #4

Supp B. Self-Efficacy Assessment of Neonatal Fellows for the Study on "Caring for Babies and their Families: Providing Psychosocial Support in the NICU" Educational Course

Supp A. Simulation Scenarios

Standardized Patient #1
Standardized Patient #2
Standardized Patient #3
Standardized Patient #4

In general, we would like to see all Fellows demonstrate:

- Empathy
- Shared decision making
- Collaboration with parents as partners
- Ensure parents' safety
- Transparent, trustworthy communication
- Empower parents, foster resiliency
- Identification of possible psychological distress with appropriate response/referral for more resources as appropriate.
- Provide culturally effective care by welcoming all equally.

Standardized Patient #1: Tamika Washington, African-American Mom with postpartum depression

Tamika Washington	African-American mother	40 years old	Baby: J'den	24 weeks, now 41 CGA	Postpartum depression (mom frustrated baby not eating well)

Family history: Mother (Tamika Washington) is a 40-year-old African American woman who gave birth to a 24 weeker unexpectedly. Her delivery was traumatic to her in that she had an emergency Cesarean delivery due to placental abruption; the abruption was likely related to the fact that she has been a long-time smoker.

Mother is married and has 3 other children at home; all were born at term and are now 2, 4 and 6 years old. Her husband has a job that is very demanding and adding to the stress of having his newest baby in the NICU. His visiting in the NICU has been limited because he has to stay home with the other kids while mom is at the NICU at night. He calls often. He's doing the best that he can, but he is barely keeping up with things.

The baby, J'den, is now 41 weeks corrected age (one week past his due date). He is on day 112 of his NICU stay. His stay has been complicated by respiratory distress which required a ventilator for a prolonged period, although he is now on room air. He required surgical closure of a PDA (patent ductus arteriosus). He had a Grade 2 intraventricular hemorrhage on one side of the brain. He was on TPN for a long time due to feeding intolerance, and he now has some cholestatic jaundice, although it is slowly resolving. He underwent laser surgery for severe retinopathy of prematurity, and he is still being followed with weekly eye exams. He has also had apnea and bradycardia, and had several "near death" experiences during his NICU stay. He is being cared for in an academic medical center NICU and there have been numerous changes of service during J'den's NICU stay, exposing mom to a large number of attendings, residents and fellows. The NICU has a peer support program, but mother declined the opportunity to be matched with a peer mentor.

J'den is still in the NICU because he is a poor feeder. Mom was pumping breastmilk but stopped about a month ago; it was too hard to keep up with all the kids and chaos at home. The baby is currently taking a premature formula (Neosure 22) but is only completing about 30% of his bottles. He has not yet completed a single full bottle. He has had the occupational therapist working with him for the last 3 weeks and he has shown minimal improvement.

Main character: Tamika Washington (background for the actor)

She displays symptoms of postpartum depression: She has a flat affect, she looks tired, she is tearful, she is anxious. When asked, she reports that she is hardly sleeping at all, she's exhausted all the time, and she doesn't have much of an appetite. When interacting with the medical team, she has been easily overwhelmed.

She hasn't been able to come to the NICU much because two of her other kids are still at home and one is in first grade. She doesn't have much support from either her husband or her family; they live in another state. She doesn't really understand why her baby can't eat well, and she is very frustrated. She just wants it all to end and have her baby be home and be normal.

One doctor had previously mentioned the possibility of her baby getting a gastrostomy tube (G-tube) and this terrified mother, so she hasn't brought it up again.

Background for fellow:

You receive sign out on the following patient:

Baby boy J'den Washington-ex 24 weeks now 16 weeks old, now doing well on RA after prolonged intubation and surgical closure of PDA, history of grade 2 IVH, history of prolonged feed intolerance and TPN dependence with some resolving cholestasis now on full feeds, had ROP requiring laser treatment, still in the NICU working on oral feeding and taking 30% po daily which is not progressing very quickly, family is nice, mostly mom visits

The Scene:

The nurse calls the Fellow to come see this mom: "Will you please call or Zoom J'den Washington's mom? I know it's your first day on service, but she needs help. In case you don't remember from signout, J'den's the former 24 weeker who is now 41 weeks; he's had like every diagnosis in the book. Anyway, she tried to give him his bottle tonight and he only took 15 ml. She's just super frustrated with his feeding and can't understand why he isn't doing better. She keeps saying she wants him home. You know we have brought up the idea of him going home on a G-tube and that didn't go over so well. So, I've run out of things to say to her. Can you please talk to her now?"

Interaction:

Fellow: [Greet the parent]

Mom: "Another new doctor? Jeesh! When am I going to see the same doctor twice? This is ridiculous. I don't understand why my baby won't take the bottle and nobody seems to be able to give me an answer. This has been going on it seems like forever and he's not improving! I'm just so sick of this NICU and all you different doctors, and I want it to be over!" Mom starts to cry.

What we would like to see the Fellow explore:

- Validate mom's feelings, acknowledge difficulty changing doctors, especially considering how long J-den has been in the NICU.
- Explore mom's bonding with baby? How do they connect?
- Highlight baby's strengths and capabilities, frame baby as strong.
- Normalize mom's emotions; acknowledge her frustration.
- Identify mother's fear.
- Identify mother's other burdens.
- Ask mom about interactions with J'den
- Discuss J'den's strengths
- Discuss G-tube vs. longer hospitalization. Discuss option of home NG feeds?
- Ask mom if she wants to wait until husband is present to have a discussion when trying to make a decision about next steps.
- Screen for PPD/PTSD (not a formal screen, just ask questions to see what symptoms she may have).

- Ask if she wants to see a social worker or psychologist while explaining their roles and how they might be able to help.
- Ask if she has peer support.
- Ask about who will be helping her out at home (assess her support systems).

Standardized Patient #2: Julie O'Donnell, White Mom with PTSD

Julie O'Donnell	White mother	24 years old	Baby: Liam	26 weeks, now 29 weeks CGA	PTSD (hx of multiple traumatic losses)

Family History: Mom is a 24-year-old primiparous White woman who gave birth by emergency Cesarean delivery after having a seizure due to maternal preeclampsia to a 1000 gm 26 weeker baby boy named Liam. Her delivery experience was traumatic, as she thought she was going to die, she was unconscious when delivered, and she didn't get to see her baby for 72 hours as she was in the ICU. Her baby is now 2 weeks old, on nasal CPAP, has a PICC line for TPN, and small enteral feeds which he is having some trouble tolerating.

Mom's social history includes that the father of her baby was shot and killed in her fourth month of pregnancy in an accidental shooting. Her father died of cancer a few months before she became pregnant. She is unemployed, without much financial resources or social support. She is, however, now in a relationship with a man who is 25 years older than her who is an alcoholic. She is estranged from her family because they didn't approve of her relationship with the father of her baby, and they didn't come to his funeral, so they aren't speaking. Because she has suffered a lot of traumatic losses recently, she is terrified that this baby will die.

Main Character: Julie O'Donnell (background for the actor)

Mom is highly anxious and agitated. She is demanding to see "the doctor." She has been hypervigilant, spending hours at her baby's bedside. She found the nasal prongs slightly askew and tried to fix them herself, and the nurse reprimanded her. She is quite angry. She complains that she thinks the nurse is neglecting her baby, not checking the baby often enough.

Background for fellow:

Sign out on this patient:

Baby boy Liam O'Donnell-ex 26 week infant 2 weeks old (?) doing well on CPAP, TPN via PICC and some trophic feeds, some stops and starts to feeds due to intolerance, some social issues here with the baby's father killed during her pregnancy, the man who visits with her is not the biologic father but is older so don't confuse with grandfather, she is estranged from her family

The Scene:

The nurse calls the Fellow to call the mother via Zoom. "Hey Doc, I've got a really difficult parent that just won't listen to me at all. She's having a meltdown over the baby's CPAP, and now she's telling me she doesn't want me to be the baby's nurse anymore. Can you please video call her and talk to her?"

Interaction:

Fellow: "Good afternoon, Ms. O'Donnell. How can I help you?"

Mom: “This nurse doesn’t even check my baby, and then when I tried to fix his CPAP myself SHE got mad at ME! I’m the one who should be getting mad at her! I don’t think she’s qualified to care for my baby. I want a new nurse. I don’t want her to ever touch my baby again.”

What we would like to see the Fellow explore:

- Explore social history to find out why she is so anxious.
- Validate mom’s feelings, acknowledge the trauma of her delivery and her baby’s hospitalization.
- Identify mother’s fear of losing baby.
- Support the mother’s care at bedside.
- Support the nurse’s care and reinforce what mother can and can’t do with baby (can’t adjust the CPAP) while also working out a plan to provide mother with reassurance.
- Support the parent’s role at the bedside by offering activities that are specific and important to be provided by parents.
- It would be a good idea to include the charge nurse in the conversation.
- Screen for PPD/PTSD (ask questions rather than do a sit-down EPDS scale).
- Ask if she has gotten any help to process her recent losses, and if she wants to see a social worker or psychologist while in the NICU while explaining their roles and how they might be able to help.
- Ask if she has peer support.
- Ask about who will be helping her out at home (assess her support systems).

Standardized Patient #3: Toma Kim, Asian Dad exhibiting feelings of anger

Toma Kim	Asian father	30 years old	Baby: Ryan, has congenital heart disease	34 weeks, now 44 weeks CGA	Anger (mom is depressed; dad is trying to hold it together)

Family History: Dad is a 30 years old, of Asian descent, and has a demanding but stable job as the CEO of a radiology practice. He and his wife have been married for 5 years and had no issues getting pregnant with their first child. His wife delivered their son, Ryan, at 34 weeks after an induction several days after premature rupture of the membranes occurred. Ryan will have a very long hospital stay because of a congenital heart defect requiring multiple surgeries. They are in month 2 of their stay with no discharge date in sight.

Main Character: Toma Kim (background for the actor)

Dad has been very involved from the moment Ryan was born. While mom recovered from her c-section, Dad spent hours in the NICU and eased back into his work schedule as mom healed. He asks a lot of insightful questions and has some base level medical knowledge due to his career in radiology. Dad visits Ryan every morning at 5 am before he goes to work and then again in the evening when he gets off work. Dad is typically pleasant and respectful to staff but lately has appeared more tense.

Mom visits during the day but goes home around 4pm each day as she is a freelance writer and works at night. She has struggled with pumping and feels inadequate as a new mom. They have a good relationship but it is strained right now with Mom slipping into depression and though she visits daily she seems detached and unable to bond with the baby. She has visited with her OB and is on antidepressants, but they don't seem to do much good. Dad is trying to "be strong" and hold things together dealing with insurance, his job, fielding all the questions from family and friends.

Background for fellow:
Sign out fellows will get:

Ryan Kim is an ex-32 week infant who is now 2 months old (40 weeks CGA). He has a postnatally confirmed, prenatally diagnosed DORV with Tet -like physiology who has had significant issues with oral feeding, intermittent feed intolerance, and weight gain, we are attempting to grow him more prior to surgery. A little background on parents: Nice parents, Dad is a radiologist, both visit frequently.

The Scene:

Dad was alone in the unit the night before (Mom had already gone home for the evening), holding Ryan before tucking him in at night, when the baby's pulse ox alarm kept going off. Ryan was fine; it was just that the sensor needed to be changed. Dad overheard a nurse say to Ryan's nurse, "Don't you need to do something about that alarm?" to which Ryan's nurse said, "Oh he's fine. I just need to change the sensor. I'll do it later."

Dad looked up and the nurse put her finger up at him to "wait a minute"...several minutes passed, the alarm kept going off and Ryan started to cry. The nurse was with another baby and unable to assist dad. The charge nurse walked by and Dad said, "You need to do something about this. Take the baby and fix

the alarm. I'm so sick of you all not paying attention to him!" Dad then stormed out of the unit because he got so angry with the nurse.

Dad has now requested (the next day) to see the doctor in hopes of determining a schedule or timeline of when Ryan might be able to come home. The day nurse has filled the Fellow in on this episode and told the Fellow to “Watch out, the guy’s kind of losing it!”

Interaction:

Fellow: “Good afternoon, Mr. Kim. How can I help you?”

Dad: “I want Ryan home. Now. Full stop. I’m just so goddamn sick of this place and the neglect and the inconsistent care by the nurses. My wife and I can do more at home than you guys are doing for Ryan here. So tell me when we can make this happen. I’m serious, I’m not screwing around with you people any longer. We’ll bring him back when he’s ready for his surgery.”

What we would like to see the Fellow explore:

- Validate dad’s feelings, acknowledge the trauma of his son’s hospitalization.
- Explore marital relationships in terms of mutual support.
- Screen for PPD/PTSD (ask questions rather than do a sit-down EPDS scale).
- Ask if he wants to see a social worker or psychologist while in the NICU.
- Ask if he has peer support or family support.

Standardized Patient #4: Angie Taylor, Lesbian Mom with PTSD

Biomom Angie Taylor, wife is Kelly	White	35 years old	Baby: Twins Chloe (doing well) & Ava (not doing well)	29 weeks, now 35 weeks CGA	PTSD (hx of difficulty conceiving, previous loss)

Family History: Bio-mom, Angie Taylor, is 35 years old and has been married for 6 years to a cisgender woman, Kelly. They have a stable income and happy home. Angie conceived by IVF and gave birth to twin girls, Chloe and Ava, at 29 weeks due to preeclampsia. Since the twins' birth, Angie visits daily after work. Kelly, who works longer hours, tries to visit every few days. At 45 days into their NICU stay, Chloe has done quite well after initially requiring breathing support on the ventilator. She is tolerating her feeds well, on nasal cannula, no PDA, no ROP, no IVH and will soon be able to attempt breastfeeding. Chloe enjoys kangarooing with both of her parents. Ava has had a very rough time; her lungs are in very bad shape and she continues to need significant respiratory support on the high frequency oscillating ventilator. Angie and Kelly were able to hold her once, but since then, Ava and has not been stable enough to be held. She had a PDA that had to be surgically ligated, has a grade III bleed on the left side, and is still on TPN for nutrition to supplement very small feeds.

The twins' moms have tried having children for 4 years, including Angie experiencing a prior miscarriage at 10 weeks, four months before successfully conceiving the twins. Following the miscarriage, Angie experienced significant anxiety about being able to conceive again and her body's ability to carry a baby to term. She experienced flashbacks of the miscarriage for two months. In order to conceive, Angie underwent two rounds of egg retrieval for IVF. Additionally, the couple has experienced financial hardship trying to conceive. Kelly bears the stress of this more than Angie as Kelly is the higher earner. Occasionally, the moms argue about paying the bills, but generally they have supported each other through the miscarriage, and now NICU stay. Since the twins' birth, Angie has had difficulty sleeping. She regularly lays awake in bed at night checking her phone for updates on the twins. Kelly is gentle with Angie but is not sure how supportive she is being.

Main Character: (background for the actor)

Angie doesn't ask many questions but takes copious notes during rounds. She occasionally goes to the unit's weekly Lunch 'n Learn sessions but seems to prefer to sit with her babies on her own. She is very quiet but has bonded with one of the nurses taking care of Ava. Her demeanor is overall quite calm and friendly. Both she and her wife are always polite to the staff but keep to themselves for the most part.

Background for fellow:

Sign out on infants: Taylor twins

Chloe Taylor-twin A 29 weeks, now 45 days old, doing well s/p short time on the ventilator, now on HFNC, full enteral feeds, starting to cue, no IVH.

Ava Taylor-twin 29 weeks, now 45 days old, has been much more ill with significant lung disease and is on HFOV with somewhat stable settings, has a history of NEC with ostomy placement, left grade 3 IVH and only tolerates small volume feeds

The Scene:

Angie is engaging in skin-to-skin kangarooing Chloe and receives a call from Ava's bedside nurse who gives her a report that Ava is doing wonderfully and has even weaned on her vent settings a bit. Angie tells the nurse she'll be over to Ava's pod in half an hour to change Ava's diaper, take her temperature, and do the sponge bath.

Half hour later, Angie has tucked Chloe in for a nap and gathers her things to visit Ava, feeling hopeful and happy. When Angie steps into Ava's pod she sees that Ava is sedated, has a chest tube, and now an IV in her scalp. She comes to Ava's bedside and sees the doctor in the room, finishing up after all the procedures. Tears stream down mom's face. Mom is so torn between the joy she feels in being able to bond with Chloe through kangarooing and being so involved in her care, while blaming herself for Ava's condition. She has been afraid to get close to Ava out of fear Ava may die, which in her mind would be her fault.

Interaction:

Mom comes into Ava's room and starts crying upon seeing her. She also sees the Fellow in the room who is finishing up after inserting the chest tube. She almost whispers to the fellow: "And you couldn't even let me know an emergency was taking place while I was just over there with my other baby? Is Ava going to make it?"

Fellow says: "Oh Ms. Taylor. You can see I'm just finishing up with Ava. She surprised us with blowing one of her lungs out. But she's ok."

What we would like to see the Fellow explore:

- Identify mother's fear of losing baby.
- Explore history to find out why mom is so fearful of Ava dying.
- Validate mom's feelings, acknowledge the trauma of her fertility journey, delivery and her twins' hospitalization.
- Evaluate the support she gets from her partner.
- Screen for PPD/PTSD (ask questions rather than do a sit-down EPDS scale).
- Ask if she has gotten any help to process her previous loss, and if she wants to see a social worker or psychologist while in the NICU.
- Ask if she has peer support.
- Highlight what she can do to feel connected and to enhance Ava's experience.

Supp B. Self-Efficacy Assessment of Neonatal Fellows

Answer the following on a scale of 1-6.

1	2	3	4	5	6
**Strongly Disagree**	**Disagree**	**Somewhat Disagree**	**Somewhat Agree**	**Agree**	**Strongly Agree**

1. **Communication Skills**
   1.1. I am a good listener.
   1.2. I am aware that we all have implicit biases that can impact service delivery.
   1.3. It's important to me to keep working to become aware of my own biases to keep them from affecting the care I give to families.
   1.4. I am able to communicate with NICU parents in a way that they will not feel judged.
   1.5. I know the impact that previous traumas may have on NICU parents' behaviors.
   1.6. I know how to comfort distressed parents even when I am busy taking care of their sick child.
   1.7. I can create collaborative relationships with families.
   1.8. I [know how to?] involve parents in decision-making about their baby's care.
2. **Emotional Support of NICU Parents**
   2.1. I am sensitive to the range of emotions that NICU parents may feel.
   2.2. I am aware that the NICU can be a very traumatic experience for parents.
   2.3. I am unlikely to take NICU parents' anger as being personally directed at me.
   2.4. I play an important role in making the NICU experience less traumatic and distressing for parents.
   2.5. Providing emotional support to NICU parents is just as important to me as taking care of their baby.
   2.6. I can recognize factors in NICU parents that increase their risks for postpartum depression and post-traumatic stress disorder.
   2.7. I can recognize the signs and symptoms of postpartum depression and post-traumatic stress disorder in NICU parents.
   2.8. I know how to do a brief assessment of how the parents of babies in my care are coping.
3. **Infant Mental Health**
   3.1. I am aware that babies in the NICU can experience a great deal of stress.
   3.2. I am aware that separating babies from their parents when they are in the NICU is traumatic for both babies and their parents.
   3.3. I am aware that the distress that babies and/or their parents may experience in the NICU can negatively affect the quality of parent-infant interaction.
   3.4. I have several strategies for how to assess parent-infant interaction and their relationship.
   3.5. I have the skills necessary to involve parents in the developmental care of their NICU baby.
   3.6. I have strategies to recommend to parents for how they might mitigate their baby's pain in the NICU.
   3.7. One of my roles is to empower parents to become confident and competent in the care of their baby.
   3.8. The support I give to parents at the bedside will carry over into an improved relationship with their baby.

Additional immediate post-course questions:

1. This simulation experience improved my knowledge about how to better provide emotional support to NICU parents and families. [Likert scale]

    a. Please explain why the program did or did not improve your knowledge about how to better provide emotional support to NICU families.
2. Participation in these simulations will change my practice. [Likert scale]
    a. Why will or will not taking this course change your practice?
3. I would recommend this simulation experience to other NICU colleagues. [Likert scale]
    a. Why would you or would you not recommend this course to other NICU colleagues?
